# Editorial: Reimagining roles and identity in the era of human - AI collaboration, volume II

**DOI:** 10.3389/fpsyg.2026.1965573

**Published:** 2026-09-18

**Authors:** Xi Chen, Ivan Wen, Qixing Qu, Wenjing Chen

**Affiliations:** 1 Management Science Department, School of Business and Tourism Management, Yunnan University, Kunming, China; 2 University of Hawai'i at Manoa Shidler College of Business, Honolulu, HI, United States; 3 School of International Trade and Economics, University of International Business and Economics, Beijing, China; 4 School of Economics and Management, Beijing University of Posts and Telecommunications, Beijing, China

**Keywords:** AI identity, digital self, human–AI symbiosis, identity transformation, symbiotic relations

This editorial introduces the second volume of the Research Topic *Reimagining roles and identities in the era of human–machine collaboration*. Artificial intelligence (AI) no longer merely assists human activity in digital environments, it actively intervenes in and reconfigures it. As AI moves from instrumental tool to affective, social interaction partner, it forces a reckoning with the boundaries of identity and social role that earlier frameworks took for granted (Brandtzaeg et al., 2022). The studies gathered here make a shared argument: human–AI collaboration is not a neutral technological upgrade but an active experiment in reconstructing self-perception, social relations, and behavior. Understanding it requires more than a single disciplinary lens, the behavioral logic and ethical stakes are too entangled to separate. We draw out five main findings that, together, define the contours of this experiment.

First, the design of virtual-persona interaction is not incidental, it is a primary lever shaping users' emotional bonds with AI. Realism does not scale linearly with trust: high realism builds attraction and trust, but medium realism can backfire into an “uncanny valley” response (Tao et al.). In e-commerce livestreaming, intelligent virtual hosts can build initial trust and value co-creation, but only within the limits set by users' technology anxiety (Zhong et al.). More strikingly, users are not passive recipients of these designs, they actively construct virtual parents with emotional-support functions through generative AI chatbots, channeling real kinship trauma into a dynamic process of resilience communication (Li et al.). These findings leave little doubt that human–computer interaction design is now doing real psychological and relational work, not merely functional work.

Second, AI's acceptance in consumption contexts is far more conditional than technology optimists assume. Labeling content as AI-generated is not a neutral disclosure, which can suppress self-expression by amplifying perceived eeriness and risk, moderated by product type (Cheng and Nam). Trust in AI livestream hosts is not general but transactional: it materializes through functional pathways only when utilitarian motivation is strong, while human hosts retain the upper hand through relational trust (Lin et al.). Perhaps most tellingly, creators

themselves do not trust audiences to accept AI involvement, they engage in “AI disguise,” concealing AI's role through invisible labor to protect their own credibility, a workaround that quietly produces new inequality among creators (Su et al.). Acceptance of AI, in other words, is being manufactured as much as it is earned.

Third, AI's behavioral effects cut both ways, and the studies here refuse to soften that tension. Anthropomorphic AI design boosts value co-creation intentions through social presence, but that gain erodes as users' sense of control increases (Ye et al.). Generative AI simultaneously helps and hollows out student entrepreneurship, strengthening performance through self-efficacy while undermining it through fears of substitution (Yu et al.). And in continued-use decisions, seduction is winning out over fear: pull effects currently outweigh push effects, with digital literacy dampening fear but amplifying seduction (Xia et al.). This is not a story of steady, comfortable adoption, it is one of persistent ambivalence.

Fourth, perceived AI attributes are actively reshaping how content circulates and who gets credit for it. An AI source label is enough to discount perceived creative effort, and with it, users' motivation to intervene in algorithmic curation (Lim et al.). Yet AI co-creation is not uniformly devalued which can raise perceived novelty and usefulness above what human collaboration achieves, making it a stronger driver of co-creation intent (Liu et al.). Even in high-stakes settings like policy dissemination, where digital human hosts still lag behind real hosts in effectiveness, that gap narrows substantially once anthropomorphism and credibility endorsement are engineered in Chen et al. Perception, not capability alone, is doing much of the work here.

Fifth, AI is not simply a source of convenience, it is a generator of anxiety that operates at both the individual and organizational level, and this volume does not shy away from naming it. Emotional cues like emoticons only move brand evaluation under specific cognitive conditions, not universally (Yoon). Emotional trust in AI, once treated as a monolith, in fact fractures into four distinct dimensions: perceived authenticity, emotional validation, emotional recognition, and symbolic connection (Briones-Llamoctanta et al.). And beneath the optimistic surface narrative of workplace AI adoption lies a harder truth: “algorithmic anxiety,” rooted in trust rupture and identity crisis, is actively corroding the psychological contract between employees and employers (Möhlmann et al., 2021; Shekhar and Saurombe).

Taken together, these studies make clear that the behavioral landscape of human–AI collaboration is neither settled nor simple, it is contested terrain, marked by real dialectical tension between benefit and harm, trust and suspicion, empowerment and anxiety. This is precisely the terrain the volume's central proposition demands we confront: technology does not just extend human capability, it forces an ongoing interrogation of what human capability, and human identity, actually mean (Baskerville et al., 2020). The evidence assembled here should be read as a call to action, not just a record of findings. Future research must go further, interrogating the power dynamics (Kellogg et al., 2020), cultural variation, and long-term effects (Glikson and Woolley, 2020) that remain underexamined and design must follow suit, building the inclusive, reflective technological ecosystem (Jobin et al., 2019; Mittelstadt, 2019) needed to ensure that human agency and the meaning of human connection survive the digital-intelligent wave, rather than being quietly displaced by it.
